# Travis County Medical Society

**Published:** 1889-03

**Authors:** B. F. Church

**Affiliations:** Secretary


### SOCIETY NOTES.

### TRAVIS COUNTY MEDICAL SOCIETY.

Stated meeting held in Austin, February 21st, 1889.

Dr. Duffau in the chair. In absence of the paper on erysipelas, which was to have been read at this meeting, Dr. M. A. Taylor was invited to make some remarks upon that subject. He thoroughly reviewed the clinical history, pathological character and treatment of the disease. He thought the cause a specific one, and that germicides should be used externally; preferred hyposulphite of soda, 20 to 30 grains to the ounce of water, or a weak solution of permanganate of potassa. He has no faith in the efficiency of caustics to check the spread of the inflammation when applied to the healthy skin near the line of demarcation. Internally, after the action of a saline cathartic, he relies principally upon the mur. tr. of iron in large doses.

Dr. J. W. McLaughlin said the question was settled beyond controversy, that erysipelas is a contagious, infectious disease, and laid stress upon the necessity of the unsparing use of disinfectants by practitioners, while in attendance upon puerperal women, or, better still, abandon their obstetrical practice while attending cases of erysipelas. The tincture of the chloride of iron, and quinine with some germicidal application to the seat of the disease; preferably, naphthalin, 20 or 30 grains to the ounce, in some ointment, is the specific treatment.

Dr. J. M. Litten remarked that he fully agreed with the gentlemen who spoke before him, as to the treatment of erysipelas. His favorite local remedy was oxide of zinc, sprinkled on dry; renew the application as it becomes moistened by the exudations; keep up the powers of the patient by good food and stimulate if indicated.

Dr. Smith called attention to one condition in this affection, which writers generally fail to mention; e. g., enlarged lymphatic glands.

The simple form where the deeper structures are not involved, usually yields kindly to treatment, but where erysipelas occurs as a complication, or in cachectic conditions, a result of surgery or other traumatism, or involves deep seated organs, the prognosis is not favorable. Externally, he applies the following ointment to the affected parts, and claims that it will ease the pain, reduce the inflammation and swelling in a few hours :

| | | |
|---|---|---|
| ℞ | Acid, boracici | ℥ss. |
| | Tr. opii deod. | ʒij. |
| | Ext. bellad. fld | ʒj. |
| | Ol sassafras | |
| | Ol camphoræ | aa gtt. xvj. |
| | Ongt. Zinci ox. | ℥ij. |
| | M. et fiat ongt. | |

Internally, he restores the secretions, stimulates the appetite for food by the judicious use of the vegetable alteratives. No specifics; he gives the sulphide of arsenic 1-55 of a grain, t. i. a. and the syr. of hypophospite of soda in drachm doses.

He regards good feeding as the principal part of the treatment; not given in small quantities at short intervals, but a suitable kind in as large proportion as can be managed at regular meal times. Thinks the principal virtue of mur. tr. of iron, is its hydrochloric acid.

Dr. Bennett thought good results may be obtained from the use of fluid extract of jaborandi in 20 or 30 minim doses. When the inflammation extends to the cellular tissue, he advised the deep injection of bichloride of mercury near the line of demarcation, to stop the spreading. He had checked it by painting the surface with a strong solution of nitrate of silver.

Dr. Tyner thought that all cases of erysipelas were traumatic, in the sense that the specific cause must enter the body from the outside. The deeper tissues of the orbital cavities are occasionally invaded; this complication may be detected by deviation of the eye ball or other symptoms indicating extra-ocular pressure; in such cases incisions should be made.

Dr. Denton was appointed to write a paper for the next meeting, March 7th, to be entitled "The Menopause."

The society adjourned.

B. F. CHURCH, M. D., Secretary.

